# First detection of bovine coronavirus in Yak (*Bos grunniens*) and a bovine coronavirus genome with a recombinant HE gene

**DOI:** 10.1099/jgv.0.001254

**Published:** 2019-04-01

**Authors:** Qifu He, Zijing Guo, Bin Zhang, Hua Yue, Cheng Tang

**Affiliations:** ^1^​ College of Life Science and Technology, Southwest University for Nationalities, Chengdu, PR China; ^2^​ Key Laboratory of Qinghai-Tibetan Plateau Animal Genetic Resource Reservation, Chengdu, PR China

**Keywords:** Yak, bovine coronavirus, molecular prevalence, genome, isolation, HE gene recombination

## Abstract

The yak (*Bosgrunniens*) is a unique domestic bovine species that plays an indispensable role for herdsmen in the Qinghai–Tibet Plateau. Here, 336 diarrhoeic samples were collected from yaks on 29 farms in the Qinghai–Tibet Plateau from 2015 to 2017. Approximately 69.05 % (232/336) of the diarrhoeic samples were assessed as bovine coronavirus (BCoV)-positive by RT-PCR assay, and most of the detected strains showed a unique evolution based on 40 spike (S), nucleocapsid (N) and haemagglutinin-esterase (HE) gene fragments. Notably, the 12 complete S genes detected shared 1 identical amino acid mutation (E121V) in the S1 subunit compared with the other 150 complete S genes in the GenBank database. Furthermore, a BCoV strain (designated YAK/HY24/CH/2017) was isolated from one diarrhoeic sample (virus titre : 10^8.17^TCID_50_ ml^−1^), and a phylogenetic analysis based on complete genome sequences revealed that strain YAK/HY24/CH/2017 has the closest genetic relationship with the BCoV prototype strain Mebus. Interestingly, 2 significant characteristics were observed in the genome of strain YAK/HY24/CH/2017 :  (1) the strain had 26 unique amino acid variations in the S gene compared with the other 150 BCoV S genes in the GenBank database and (2) a recombination event was identified between the esterase and lectin domains of the HE gene. In conclusion, this study revealed the high prevalence of BCoV in yaks in the Qinghai–Tibet Plateau. To the best of our knowledge, this is the first description of the molecular prevalence of BCoV in yaks and of a BCoV genome with an HE gene recombination.

## Introduction

Bovine coronavirus (BCoV) is a causative agent of diarrhoea in neonatal calves, winter dysentery in adult cattle and respiratory tract illness in cattle of all ages, leading to serious economic losses [1]. In addition to infecting cattle, BCoVs have the potential for interspecies transmission to wild ruminant animals [2–4], including caribou [5], white-tailed deer [6], waterbuck [6], elk [7], alpaca [8], giraffe [4] and sambar deer [9], given that wild ruminant coronaviruses (CoVs) are biologically, antigenically and genetically similar to bovine CoVs from domestic cattle [8–10]. Additionally, a CoV strain named HEC 4408 was isolated from a diarrhoeic sample from a child in Germany, the genome of which revealed that the strain was more closely related to BCoVs than to other human (H)CoVs, indicating that the strain might have originated from BCoV [11]. Dual pneumoenteric tropism is a common feature of BCoV [12]; a recent study also suggested that human nasal mucosa can temporarily carry BCoV RNA after exposure to virus-shedding calves [13]. These results indicate that the public health impact of BCoVs needs to be further investigated.

To date, there are 38 BCoV genomes (including 23 BCoV genomes from cattle and 12 BCoV-like genomes from ruminants, as well as 3 BCoV-like genomes from humans) in the GenBank database. Host species-specific mutations involving deletion in the variable region of the S1 subunit (from amino acid 543 to amino acid 547) were detected in giraffe CoV [14], but the genomic features of CoV detected in other wild ruminants could not be discriminated from BCoV [2, 10]. The BCoV virion contains five structural proteins: the spike (S) protein, the haemagglutinin-esterase (HE) protein, the nucleocapsid (N) protein, the transmembrane (M) protein and the small envelope (E) protein [15]. The S protein is important for viral entry and pathogenesis, and it is divided into the S1 (N-terminus) and S2 (C-terminus) subunits. S1 is the globular subunit; it is responsible for virus binding to host cell receptors and the induction of neutralizing antibody expression and haemagglutinin activity. S2 is the transmembrane subunit; it is required to mediate the fusion of viral and cellular membranes [16]. The HE protein has been shown to possess an esterase receptor-destroying activity that may be important for virus entry, and it may also serve as a second viral attachment protein (in addition to the large S protein) for the initiation of infection [17]. The evolution of CoVs is facilitated by their high mutation rates, inter- and intra-host selection and recombination frequency, as well as by selection pressure on genetic diversity and genetic drifts during transmission bottlenecks [18–20], all of which may lead to changes in the virulence, tissue tropism and host range of these viruses [21, 22].

The yak (*Bos grunniens*) is a unique long-haired bovine species that belongs to the genus *Bos* within the family *Bovidae* [23]. There are over 14 million yaks worldwide, which are distributed in the high-altitude regions (above 2500–6000 m) of China, India, Nepal, Pakistan, Kyrgyzstan, Mongolia and Russia and are mainly located in the Qinghai–Tibet Plateau in China. The yak is an indispensable animal for the local people in the Qinghai–Tibet Plateau, providing meat, milk, skins, transport and fuel (faeces) [24]. Diarrhoea is a common disease in yaks; however, there is still limited information on diarrhoea pathogens in these animals because of the harsh natural environment, inconvenient travel and fragmented veterinary services in the Qinghai–Tibet Plateau [25]. Therefore, the aim of this study was to investigate the prevalence and molecular characteristics of BCoV in yaks in the Qinghai–Tibet Plateau.

## Results

### Detection of BCoV in yaks

**Fig. 1.**
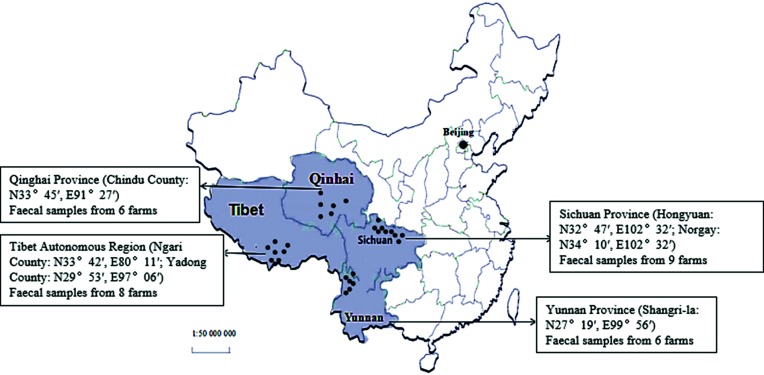
Map of China showing the geographical distribution of the sample collection sites. The small black dots on the map indicate the provinces from which the samples in this study were collected. The large black dot indicates the capital of the People’s Republic of China, Beijing.

Among the 336 diarrhoeic samples, 232 (69.05 %) were detected as BCoV-positive by RT-PCR. Notably, BCoV was found in diarrhoeic samples from all 29 farms in all 4 provinces. These provinces are the main yak-producing areas in China, and the geographical distance between the two furthest farms is >1500 km (Fig. 1). The detection rates were 68.33 % 71.67 % 69.23 and 66.67 % in the Tibet Autonomous Region, Qinghai Province, Sichuan Province and Yunnan Province, respectively.

### Phylogenetic analysis of partial S, HE and N genes of BCoV

**Fig. 2.**
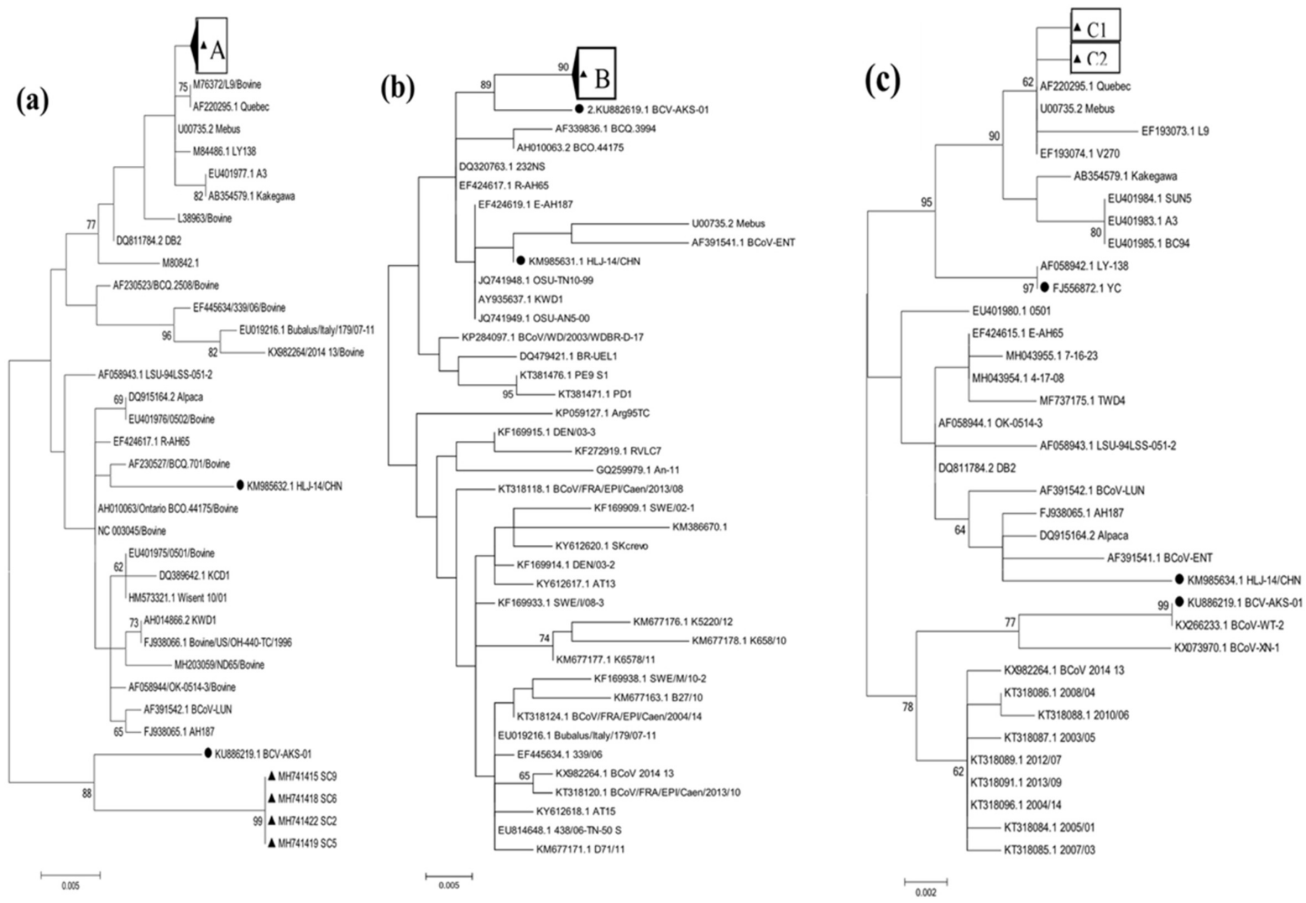
Maximum-likelihood trees based on the 40 partial S, HE and N genes in this study. (a) Partial S gene (941 bp); ‘A’ represents the strains from this study listed under accession nos MH741304–MH741343. (b) Partial HE gene (775 bp); ‘B’ represents the strains from this study listed under accession nos MH741384–MH741414, MH741416, MH741417, MH741420, MH741421 and MH741423. (c) Partial N gene (645 bp); ‘C1’ represents the strains from this study listed under accession nos MH741364–MH741383. ‘C2’ represents the strains from this study listed under accession nos MH741344–MH741363. The BCoV strains from this study are marked with a black triangle, and the other BCoV strains from China are marked with a black circle.

We simultaneously amplified the S, HE and N gene fragments from a total of 40 BCoV-positive samples (10 BCoV-positive samples from each province), and they were submitted to the GenBank database with accession numbers MH741304–MH741423. A phylogenetic analysis of the 40 partial S gene sequences showed that all 40 yak CoV strains detected in this study were most closely related to strain AKS-01, which was isolated from a Chinese dairy cow (GenBank accession number KU886219.1), and they were located in an independent sub-branch (Fig. 2a). A phylogenetic analysis of the 40 partial HE gene sequences showed that 36 of the strains were most closely related to strains BCV-L9 and Quebec (GenBank accession numbers M76372 and AF220295.1, respectively), and they were clustered into an independent sub-branch; the remaining 4 strains were clustered into a large independent branch with strain AKS-01, which was determined in China (GenBank accession number KU886219.1) (Fig. 2b). A phylogenetic analysis of the 40 partial N gene sequences showed that all 40 strains in this study were most closely related to strains Quebec and Mebus (GenBank accession numbers U00735.2 and AF220295.1, respectively), which were both determined in the USA (Fig. 2c).

### Virus isolation

Through three passages after inoculation, cytopathic effect (CPE) was found in one out five cell cultures, characterized by enlarged, rounded and densely granular cells in clusters. The strain (YAK/HY24/CH/2017) was successfully isolated from a diarrhoeic faecal sample collected from a 2-month-old calf in Sichuan Province in July 2017. Stable CPEs were observed from passages 6–10, after which the virus was purified by plaque purification. BCoV-specific cytoplasmic fluorescence was detected in HRT-18 cells by indirect immunofluorescence tests and coronavirus particles were observed using transmission electron microscopy (data not shown). The virus titre of the isolated strain was calculated as 10^8.17^TCID_50_ ml^−1^ according to the Reed*–*Muench method.

### Genomic characterization of isolated strain YAK/HY24/CH/2017

#### Overview of isolated strain YAK/HY24/CH/2017

**Fig. 3.**
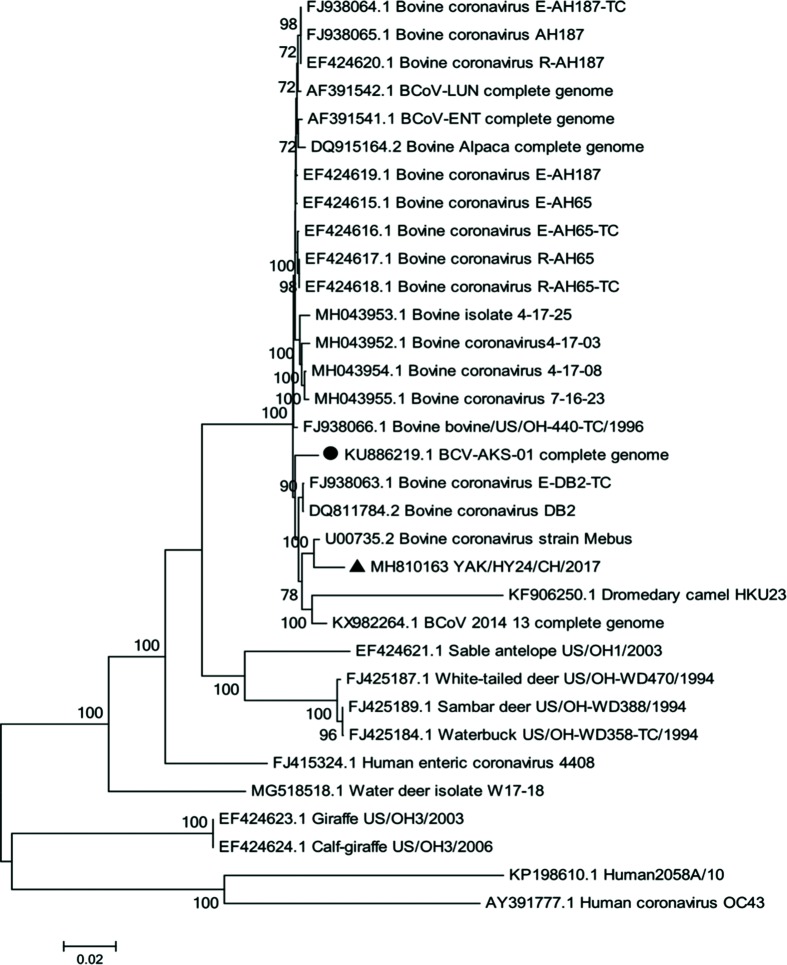
Maximum-likelihood tree based on complete coronavirus genome sequences. Strain YAK/HY24/CH/2017 from this study is marked with a black triangle, and the other BCoV strain determined from China is marked with a black circle.

The complete genome of the strain YAK/HY24/CH/2017 isolated in this study was successfully sequenced, and the sequence was submitted to the GenBank database with accession number MH810163. The linear genome is 31 032 nt in length with a G+C content of 37.1 %. The complete genome of the strain shares 84.7–98.4 % nt identity with all 38 BCoV genome sequences in the GenBank database. A phylogenetic analysis of the complete genome sequences revealed that strain YAK/HY24/CH/2017 is clustered into an independent sub-branch with the BCoV prototype strain Mebus (Fig. 3).

#### Complete S gene of strain YAK/HY24/CH/2017

**Fig. 4.**
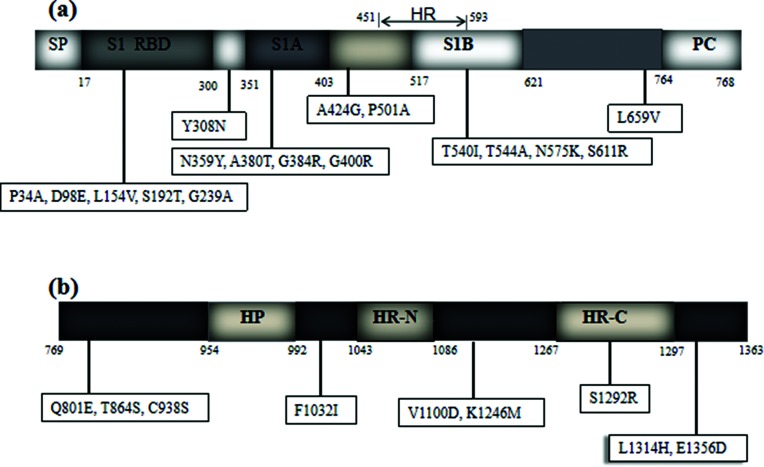
Amino acid variants of the complete S gene of strain YAK/HY24/CH/2017. The figures in the boxes indicate amino acid change sites in strain YAK/HY24/CH/2017 compared with the other 150 available BCoV S sequences in the GenBank database and 12 BCoV S sequences cloned by our laboratory (MH810151–MH810162). (a) Amino acid variants in the S1 subunit of strain YAK/HY24/CH/2017. (b) Amino acid variants in the S2 subunit of strain YAK/HY24/CH/2017. HP, the first hydrophobic domain of the S2 subunit; HR, hypervariable region; HR-N and HR-C, the heptad repeats; PC, proteolytic cleavage region; RBD, receptor-binding domain; S1A and S1B, the immune reactive domain; SP, signal peptide.

The complete S gene of strain YAK/HY24/CH/2017 is 4092 bp in length and encodes 1363 amino acids. The complete S gene of this strain shares 95.1–98.5 % nt identity (94.7–98.8 % aa identity) with all 162 available BCoV S sequences in the GenBank database (including 12 BCoV S sequences cloned by our laboratory, GenBank accession numbers MH810151–MH810162). A phylogenetic analysis of the complete S gene amino acid sequences showed that strain YAK/HY24/CH/2017 is most closely related to the prototype strain Mebus. Further analysis revealed that strain YAK/HY24/CH/2017 has 17 unique amino acid variations in the S1 subunit (Fig. 4a) and 9 unique amino acid variations in the S2 subunit (Fig. 4b) compared with all 162 available BCoV S sequences.

#### Complete HE gene of strain YAK/HY24/CH/2017

**Fig. 5.**
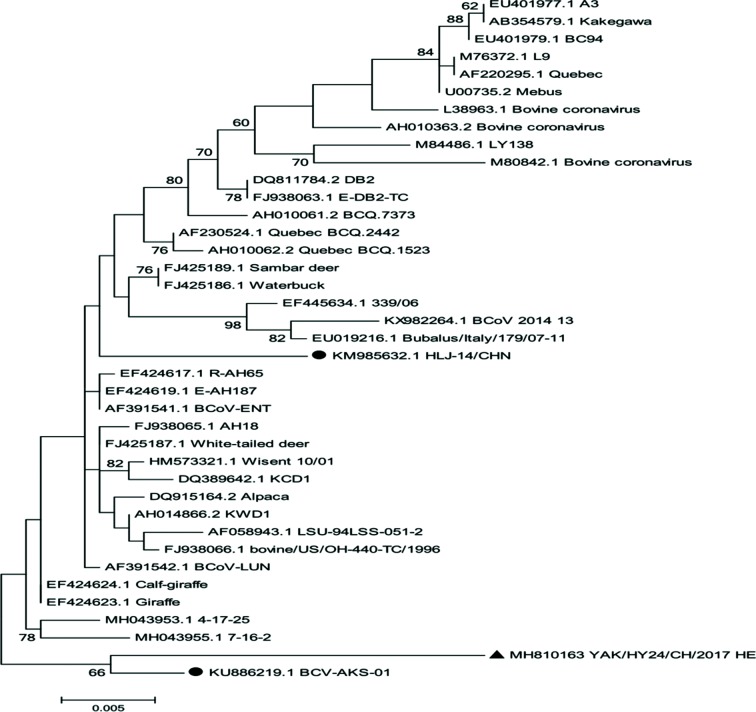
Maximum likelihood tree based on the deduced 424 aa sequences of the complete HE protein of strain YAK/HY24/CH/2017. The BCoV strain from this study is marked with a black triangle and the other BCoV strains determined from China are marked with a black circle.

**Fig. 6.**
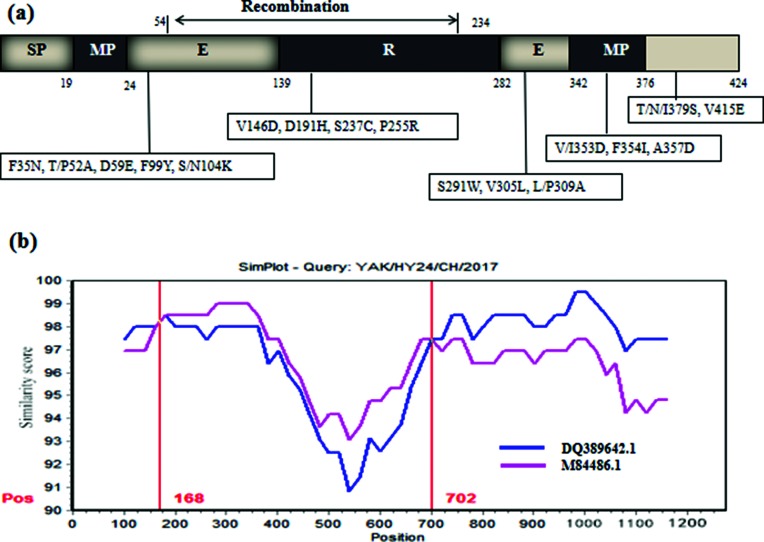
Sequence and recombination analysis of the HE gene of strain YAK/HY24/CH/2017. (a) Amino acid variants in the HE gene of strain YAK/HY24/CH/2017. The figures in the boxes indicate the amino acid change sites in the HE gene of strain YAK/HY24/CH/2017 compared with all other 107 available BCoV HE sequences in the GenBank database. (b) Recombination analysis of the HE gene of strain YAK/HY24/CH/2017 using SimPlot 3.5.1. A nucleotide (nt) identify plot comparing a 1275 bp fragment of strain YAK/HY24/CH/2017 with BCoV strains KCD1 (GenBank accession number: DQ389642) and LY138 (GenBank accession number: M84486) is shown. The putative recombination region was located at 168–702 nt. The vertical axis indicates the similarity (%) of nucleotide sequences between the query strain and other reference strains. The horizontal axis indicates the nucleotide positions. SimPlot analysis was performed using a window size of 200 nt and a step size of 20 nt.

The complete HE gene of strain YAK/HY24/CH/2017 is 1275 bp in length and encodes 424 amino acids. This complete HE gene shares 95.2–97.6 % nt identity (94.6–97.9 % aa identity) with all 107 available BCoV HE gene sequences (including 92 complete HE genes from cattle and 12 complete HE genes from ruminant animals, as well as 3 complete HE genes from humans). A phylogenetic tree of the complete HE gene amino acid sequences showed that strain YAK/HY24/CH/2017 is clustered into an independent branch with strain AKS-01, which was determined in China (GenBank accession number KU886219.1) (Fig. 5). Further analysis revealed that strain YAK/HY24/CH/2017 has 17 unique amino acid variations compared with all 107 available BCoV HE gene sequences (Fig. 6a). Additionally, there are 4 identical amino acid variations (D/G66S, N147T, F/S181V and V407L) in strains YAK/HY24/CH/2017 and AKS-01 compared with the other 106 available BCoV HE gene sequences, and there is 1 identical amino acid variation (V188A) in strain YAK/HY24/CH/2017 and 4 strains determined in Vietnam (GenBank accession numbers MH203060–MH203063) compared with the 103 other available BCoV HE gene sequences.

Notably, a recombination event was predicted in the HE gene of strain YAK/HY24/CH/2017 when using Simplot 3.5.1 and Recombination Detection Program (RDP) 4.0 with the RDP, GeneConv, Chimaera, MaxChi, SiScan and 3Seq methods (recombinant score=0.613). The recombination region identified using RDP 4.0 was predicted to be located at nt 150–724 of the fragment (beginning breakpoint 99 % confidence interval: nt position 1–524 in the fragment; ending breakpoint 99 % confidence interval: position 544–869 in the fragment). The putative major parental strain is strain KCD1, which was determined in the Republic of Korea (GenBank accession number DQ389642.1), and the possible minor parental strain is strain LY138 (GenBank accession number M84486.1). Moreover, using SimPlot 3.5.1, the recombination region of the putative parental strains was mapped to nt 168–702 (Fig. 6b). Although the recombination breakpoints predicted by RDP 4.0 and SimPlot are different from one another, both programs showed that the recombination breakpoint is located between the esterase and lectin domains in HE. Further analysis revealed that a recombination event was also predicted in the HE gene of strain AKS-01, and the recombination region of strain AKS-01 is consistent with that of strain YAK/HY24/CH/2017 in this study.

## Discussion

### Prevalence of BCoV in yaks in China

The yak is a unique free-grazing bovine species in the Qinghai–Tibet Plateau [24]. Diarrhoea, as a severe disease in yak, results in high mortality. However, there are still limited epidemiological data on diarrhoea pathogens in yak [25]. In this study, approximately 69.05 % of diarrhoeic samples were detected as BCoV-positive by RT-PCR, which indicates that BCoV plays an important role in yak diarrhoea in the Qinghai–Tibet Plateau. Furthermore, BCoV-positive samples were distributed across all 29 farms located in the 4 main yak-production areas in China (the geographical distance between the two furthest farms is >1500 km), which suggests that BCoVs have been circulating widely among yaks in China. Although the epidemiological data for BCoV in cattle in China are still not clear, the presence of BCoV in dairy cattle in China has been confirmed (GenBank accession numbers KU886219.1 and KM985631). Thus, it is possible that the BCoV in yaks was transmitted from BCoV in dairy cows. To the best of our knowledge, this is first detection of BCoV in yak, and this finding will contribute to the diagnosis and prevention of yak diarrhoea in the Qinghai–Tibet Plateau.

Yaks, as free-grazing domestic animals in the Qinghai–Tibet Plateau, have large living areas, and the farmers have a convention of changing their grazing areas every year. Within the area in which the yaks live, there are many wild ruminants, such as wild yaks, antelopes [26], Tibetan gazelles [27] and Przewalski’s gazelles [27]. It will be interesting to investigate the prevalence of BCoV in wild ruminants in the Qinghai–Tibet Plateau. Additionally, there are long land borders between the Qinghai–Tibet Plateau in China and Nepal, India, Pakistan, Kyrgyzstan, Mongolia and Russia, and cross-border exchanges occur between the yaks in these countries. Therefore, BCoV from yaks also has the potential for transboundary transmission.

Interestingly, most of the strains detected in this study showed a unique evolution based on 40 s, N and HE gene fragments. Thus, the unique evolution of BCoV in yaks may be related to the special geographical environment of the Qinghai–Tibet Plateau – i.e. the high-altitude (average altitude of over 4000 m), low oxygen, low temperature and low atmospheric pressure – where the yak live, as well as to the characteristics of the host species [28–31].

### Characterization of complete S gene in strain YAK/HY24/CH/2017

The BCoV S protein is involved in receptor recognition, host specificity, antigenic diversity and immunogenicity [16], and mutations in this protein are associated with alterations in viral antigenicity, pathogenicity, tissue tropism and host range [16, 32]. In this study, strain YAK/HY24/CH/2017 showed 17 and 9 unique amino acid variations in the S1 and S2 subunits, respectively, compared with the other 162 available BCoV S sequences. Previous work suggested that the amino acid residues at positions 501, 540 and 544 of the S protein may be changed under positive selection, contributing to the continuous circulation of BCoV among cattle and other ruminants, and this may be a characteristic of BCoV adaptive evolution, whereas the amino acid residues at positions 359, 380, 384, 400, 938, 1032 and 1100 of the S protein are under negative selection [28]. Interestingly, unique amino acid variations were observed at three sites (P501A, T540I, and T544A) in the S gene of strain YAK/HY24/CH/2017, and these may be a means by which BCoV adapts to using yaks as a host. Moreover, seven unique amino acid variations (N359Y, A380T, G384R, G400R, C938S, F1032I and V1100D) were also observed in strain YAK/HY24/CH/2017. The biological significance of these variations in the adaptive evolution of BCoVs needs to be further investigated.

Previous work suggested that loops 10–11 of BCoV RBD probably contribute indirectly to sugar binding [32], and point mutations in the RBD may lead to decreases in the virus binding to the cellular receptor, allowing BCoV to avoid the binding of neutralizing antibodies [33]. Furthermore, mutations in the repeat region (HR1) domain of the S2 subunit in mouse hepatitis virus (MHV) affect the ability of the S1 subunit to bind to the receptor and also mediate host range expansion and tissue tropism [34, 35]. In this study, unique amino acid variations in the S1 subunit loop 11 (L154V), RBD (C938S) and HR1 (F1032I) of strain YAK/HY24/CH/2017 were observed. The biological significance of these variants warrants further investigation.

### Characterization of the complete HE gene in strain YAK/HY24/CH/2017

The HE protein of BCoV plays an important role in the infection process of the virus. To date, in the genus *Betacoronavirus*, only MHV had reportedly undergone a nonhomologous recombination event in the HE gene, and this recombination may affect its function [36]. Recombination has also been observed in the HE gene of influenza C virus and toroviruses [37, 38]. Recombination in the HE gene may be a strong driving force for the generation of strains with new genotypes, host spectra and tissue tropisms [36–38]. Interestingly, a recombination event was predicted between the esterase and lectin domains of the HE gene of the isolated BCoV strain YAK/HY24/CH/2017. To the best of our knowledge, this is the first description of a recombination event in a BCoV HE gene. Further analysis of the 40 partial HE genes cloned in this study showed that four strains (GenBank accession numbers MH741325, MH741328, MH741329 and MH741332), including strain YAK/HY24/CH/2017 from Sichuan province, were predicted as recombinant strains. Thus, the prevalence of HE-recombination BCoV strains should be monitored going forward. Additionally, an identical HE recombination event was also predicted in strain AKS-01 from a Chinese dairy cow in this study. It is possible that the BCoV strain with HE recombination found in yaks was originally transmitted from BCoV in dairy cows.

BCoV and HCoV-OC43 both use 9-O-acetylated sialic acid as a receptor. HE evolution has been marked by a progressive loss of HE receptor-binding activity through the accumulation of selected mutations in the HE lectin domain; for example, HCoV-OC43 is thought to be caused by an accumulation of aa substitutions (T114N, R177P, E178Q and F247L) in the receptor-binding region during the course of evolution [17, 39]. The R2 loop in the lectin domain (residues 176–185) plays an important role in ligand binding, so amino acid substitutions in this region may change the receptor-binding activity [39]. In this study, strain YAK/HY24/CH/2017 had 12 unique amino acid variations in the HE gene compared with all 107 known BCoV HE genes (Fig. 6). Therefore, further investigation into the impact of this unique BCoV HE protein on BCoV infection (both its receptor binding and enzyme destruction abilities) will likely be interesting.

## Methods

### Sample collection

A total of 336 diarrhoea samples from calves (aged <3 months) were collected from 29 farms between 2015 and 2017 (June to August each year). These four provinces are the main yak-producing areas in China (Fig. 1). Sixty, 60, 60 and 156 diarrhoeic samples collected from the Tibet Autonomous Region (8 farms), Qinghai Province (6 farms), Yunnan Province (6 farms) and Sichuan Province (9 farms), respectively. All samples were shipped on ice and stored at −80 °C.

### Nucleic acid extraction and cDNA synthesis

The clinical faecal samples were fully resuspended in phosphate-buffered saline (PBS) (1 : 5) and centrifuged at 10 000 ***g*** for 10 min, followed by filtration through a 0.45 µm filter. Viral RNA was extracted from 300 µl of the faecal suspension using RNAios Plus (TaKaRa Bio, Inc., Japan) according to the manufacturer’s instructions. The cDNA was synthesized using the PrimeScript RT Reagent kit according to the manufacturer’s instructions (TaKaRa Bio, Inc.) and stored at −20 °C.

### Screening for BCoV by RT-PCR

A total of 336 clinical samples were assessed for BCoV detection using specific RT-PCR assays established in our laboratory [40]. The pair of primers is targeted to Nsp10 of ORF1a and the amplicon is 230 bp and located at 13070–13 299 of strain BCoV-2014–13 (GenBank accession number KX982264.1).

### Partial S, HE and N gene sequence amplification

A total of 40 BCoV-positive samples (10 BCoV-positive samples from each province) were randomly selected from the Tibet, Qinghai, Sichuan and Yunnan provinces. These 40 samples were used to simultaneously amplify the S, HE and N gene fragments. The primer information is shown in Table 1. All amplification products were purified and cloned into the pMD19-T simple vector (TaKaRa Bio, Inc.) for sequencing.

**Table 1.** Primer sequences used to amplify and sequence the partial S, HE and N genes and complete S gene of BCoV

Target gene	Primer sequence	Size (bp)
Partial S gene	F:5′-GTTTCTGTTAGCAGGTTTAA-3′ R:5′-GTCGCATTAACCTCAACAAA-3′	641
Partial HE gene	F:5′-TGATAACCCTCCTACCAATG-3′ R:5′-AGACAGATTGCTTTAGTGGG-3′	774
Partial N gene	F:5′-TCAACCCAAGCAAACTGTCA-3′ R:5′-CTGCTTAGTTACTTGCTGTGGC-3′	643
Complete S gene	F1 : 5′-GCTGCATGATGCTTAGACCA-3′ R1 : 5′-ATACGTCGGTAAACATCTGC-3′ F2 : 5′-AACGGTTACACTGTTCAGCC-3′ R2 : 5′-GTCAACAGACCAGCCCAAAA-3′ F3 : 5′-TTGTGGAGGTAATCCTTGTA-3′ R3 : 5′-ATCGCTTCCTAAACAACCTAATACA-3 F4 : 5′-CCCCTGTATTAGGTTGTTTA-3′ R4 : 5′-TAACCTTCGCAGTGACATAC-3′ F5 : 5′-TGCAGCACAAGCTATGGAGA-3′ R5 : 5′-GAGCCAATAAATCAAAGACGAACT-3′	4092

### Virus isolation

Five BCoV-positive diarrhoeic faecal samples were used for virus isolation on HRT-18 cells as previously described [41]. The cells were harvested once the CPE exceeded 80 %. After the CPE stabilized and the virus was purified using a plaque assay, virus titration was performed in 96-well plates with 10-fold serial dilutions performed using 8 replicates per dilution. The virus titres were determined using the Reed–Muench method, and endpoints were expressed as the 50 % tissue culture infective dose (TCID_50_) ml^−1^ [42].

### Complete genome amplification of strain YAK/HY24/CH/2017

In total, 43 pairs of primers were designed to amplify the complete genome of the isolated strain YAK/HY24/CH/2017 (Table 2). All PCR products were purified and cloned into the pMD19-T simple vector (TaKaRa Bio, Inc.) for sequencing. The sequences were assembled using SeqMan software (version 7.0; DNASTAR, Madison, WI, USA).

**Table 2.** Primer sequences used to amplify and sequence the complete genome of BCoV strain YAK/HY24/CH/2017

Primer no.	Position	Primer sequence	Primer no.	Position	Primer sequence
1	1–304	F:GATTGYGAGCGATTTGCGTG R:TAGGGTTATCCAACTTCTCC	23	15235–16091	F:TATCGACTTGCGAATGAATG R:ACGCAAGCTCCAACACTCTG
2	237–916	F:TTTGAGGACGCAGAGGAGAA R:ACACCACGGTATCCTCTAAT	24	16087–16958	F:TGCGTTGTCTGCTCTTCTCA R:GCAAGACCGATAGCAAGATG
3	802–1525	F:AAGGGTGCCTACAATAAAGA R:ATACAGGATTAACAGGCAAA	25	16933–17624	F:CCGTGCAAGGACCTCCTGGTA R:GGCTTTATGCCACAAAGGG
4	1175–2313	F:TTGCTAACCCTACTGAAGAC R:ACTAGCATAACGAGGAATGT	26	17446–18499	F:ATACAGTGTCTGCCTTGGTT R:AAGTATCGGAGACAAGTGAG
5	1961–3064	F:TTATTTGGCAGTAAGTGGTC R:GCACAATACAATTTAGGAGCAA	27	18314–19271	F:TGTGGCTAAAGCTCCTCCTG R:AGCATCCATGCCATCCATAT
6	3039–4111	F:TTTATTTGATGAGGCTGGTG R:TAGCAGGATTTACAACGACT	28	19195–20009	F:CCTTTGAGCATCTGAAGCCTAT R:ACCATCCTTACGCACAGCAA
7	3939–4853	F:TTGGGCATGGTATGTCATTT R:TTTGAGCAACAGTAGCCTTA	29	19874–20589	F:CGTTTACATTTCCACGACAA R:AACGAGGATAGAAAGTCATA
8	4732–5557	F:TGGCGTGTTGTCAATAAGTT R:AATCCACGGTGTAACCAATC	30	20568–21420	F:GTTATGACTTTCTATCCTCGTT R:CAAGAAGGGAATAAACCATA
9	5272–6311	F:AAAATTGTTCAATGGCAGGAG R:TTGGGTTCTTTAGCATCACTCT	31	21026–21698	F:AAGTAAAGATGGTTTCTTTACATAC R:GAATAGCGACATCAACACTT
10	6290–7232	F:AGAGTGATGCTAAAGAACCCAAAG R:GGCAGCCAAGTGGTCAAGAT	32	21423–22425	F:AAGGGTAAACTACTTGTTAGAG R:TTGGTAGGAGGGTTATCAAA
11	7018–8224	F:ATCGCATGTCAGTTCTGCTT R:CCACAATGTTATCACCCTTC	33	22087–23563	F:AATACAATCCACCCACTGAC R:TATGACCGCAACACCCAAAA
12	7566–8587	F:TATTACTGTTGAGGCCGCTCTT R:AACTGGCATAAACGGGAAGC	34	23460–24509	F:GATGCCCTACTGCTGCTGAT R:AAATCGCTCTTACAATCAAC
13	8570–9296	F:TTCCCGTTTATGCCAGTTAT R:AATGCCAAGAAATCCACAGG	35	24186–25414	F:ACTATGGCATTGGGATACAG R:AAGAGTCAACAGACCAACCC
14	9062–9852	F:CAGAAGTGTTGCGAGAAGGA R:AGAACAAGCAGCCTCCCTAT	36	25210–26734	F:TGCCATAATGCTGCCCAATG R:ACCCTTCCTGAATAGCACCA
15	9488–10743	F:TTATGCCACGCTTTATTTCC R:AGCAGCCAATAGTGTTTCCA	37	26717–27 676	F:GTGCTATTCAGGAAGGGTTT R:AACAACCACCACATTTCTTA
16	10646–11442	F:GGGCTTTGTCTAATGGGTTT R:AACATTCACAGCAACCCACT	38	27473–28 745	F:AATGAATAGGTTACAGGAGGCA R:TTCATCAGCAGTCCAGGTGT
17	11341–12440	F:ATTCTTCTTATGTTGGCTTCTC R:CCCGCATAGGTAACATAGACAT	39	28726–29770	F:ACACCTGGACTGCTGATGAA R:AAATACCATCGTGGGAGCAA
18	12154–12881	F:GCAAGAAAGTTGGAGCGTAT R:GTACCAACAACCCAGCCTCT	40	29363–30157	F:CGCATTGTTGAGAAATAATATCTAA R:ACTTGCTGTGGCTTAGTGGC
19	12679–13293	F:AATGGGAAGATTGTTTATGC R:ACATGAACAGCTTCCATCCC	41	29713–30805	F:GACGTTCTTTTAAAACAGCCGATGG R:GTGCCTTATCCCGACTTTCC
20	13026–13926	F:TTGTGTTAAAATGTTGTGTGACCAT R:TGCCTACTAAGCCTACCTCC	42	30580–30978	F:GTCAGCGTGGTCAGAAGAAT R:GTAACTTAACATGCTGGCTC
21	13893–14991	F:GCAGACAAATTGGTGGAGGT R:GTATGGAAACACCAGCGACA	43	30555–31032	F:ACAGCGTCAGCGTGGTCAGA R:GTGATTCTTCCAATTGGC
22	14594–15422	F:AGAGGGTAGTTCAGTTGATT R:AGCACATACATTGGCTGAAA			

### Sequence, phylogeny and recombination analyses

For the genome organization analysis, putative open reading frames (ORFs) and their corresponding amino acids were predicted using the ORF Finder tool (http://www.ncbi.nlm.nih.gov/gorf/gorf.html). The homologies of the nucleotide and deduced amino acid sequences were determined using the MegAlign program in DNASTAR 7.0 (DNASTAR, Inc.). mega 7.0 was used to perform multiple sequence alignment and to subsequently build a maximum-likelihood phylogenetic tree with bootstrap support [43]. Recombination events were assessed using SimPlot software (version 3.5.1) and RDP 4.0 with the RDP, GeneConv, Chimaera, MaxChi, BootScan, SiScan and 3Seq methods [44].
